# Influence of Shade in Cocoa Agroforestry Systems on Physicochemical and Functional Characteristics of Cocoa Beans in Bonon, Central-West Côte d'Ivoire

**DOI:** 10.1155/2024/1543904

**Published:** 2024-03-06

**Authors:** Affoué Tatiana Kouassi, Adjoua Christiane Eunice Boko, Sika Hortense Blei, Djédoux Maxime Angaman, Yao Sadaiou Sabas Barima

**Affiliations:** ^1^Department of Biochemistry-Microbiology, Jean Lorougnon Guédé University, Daloa, P.O. Box 150, Daloa, Côte d'Ivoire; ^2^Interdisciplinary Research Group in Landscape Ecology and Environment, Jean Lorougnon Guédé University, Daloa, P.O. Box 150, Daloa, Côte d'Ivoire; ^3^Department of Science and Technology, Alassane Ouattara University, P.O. Box 18, Bouaké, Côte d'Ivoire; ^4^Department of Biodiversity and Sustainable Ecosystem Management, Jean Lorougnon Guédé University, Daloa, P.O. Box 150, Daloa, Côte d'Ivoire

## Abstract

Côte d'Ivoire remains the world's leading producer of cocoa beans. However, cocoa farming is now recognized as a primary cause of deforestation in the country. To combat deforestation, the Ivorian government recently advocates for agroforestry, a farming technique involving the cultivation of cocoa trees with fruit or forest trees. Yet, the impact of these associated trees and their shade on the quality of produced cocoa beans remains relatively unknown. This study is aimed at evaluating the influence of tree shade in cocoa farms on the quality of cocoa beans produced in the Bonon area. Morphological, biochemical, and functional analyses were performed on cocoa beans from shaded, partially shaded, and sunny subplots. Overall, only beans from shaded subplots showed better commercial quality. Regarding nutritional potential, results demonstrated that acidity, protein content, and vitamin C levels were influenced by shade. Low protein levels were observed in beans from sunny areas. The presence of moderate shade significantly favored good foaming power and foam stability. These findings play a key role in the perceived quality and application of these beans in the food and cosmetic industry. Moreover, these discoveries open new research perspectives in the field of food biochemistry and sustainable agriculture.

## 1. Introduction

In the dynamic socioeconomic landscape of Côte d'Ivoire, a fast-growing developing country, the economy is heavily dependent on the export of agricultural products. Among these products, cocoa occupies a prominent place [1]. Cocoa (*Theobroma cacao* L.) is indispensable to the confectionery industry as a primary source of cocoa beans [2], but it also produces by-products such as the pod and shell, which have found substantial applications in the cosmetics and animal feed industries. The introduction of cocoa cultivation in Côte d'Ivoire towards the end of the 19th century marked the start of a remarkable agricultural development. Since independence, the nation has experienced phenomenal growth in cocoa cultivation, consolidating its status as the world's leading cocoa producer for more than four decades, with an impressive production volume of around 2.01 million tonnes per year [3]. However, this impressive agricultural success is not without its problems. Côte d'Ivoire's cocoa sector is facing major environmental and socioeconomic problems, not least the growing pressure on the country's land and forest resources. Historical data paints a worrying picture: the forest area, which covered more than 16.5 million hectares at the beginning of the 20th century, has shrunk by an alarming 80%, leaving only around 3.4 million hectares in 2015 [4]. This drastic reduction is a striking indicator of the environmental cost of agricultural expansion, further exacerbated by the growing global demand for cocoa [5]. This trend points to an inevitable escalation in the pressure on available agricultural land. In response to these impending challenges, Côte d'Ivoire is being forced to explore and adopt sustainable farming practices. Among these, agroforestry has emerged as a promising strategy. This integrated approach to combining forestry and agriculture, agroforestry, presents a symbiotic relationship where trees and crops can thrive, achieving a balance between agricultural productivity and ecological conservation [6]. However, the introduction of agroforestry and shade management within these systems brings its own complexities. According to CIRAD [7], while the shade provided by species associated with cocoa trees has advantages such as regulating the microclimate and adding organic matter, it can also have adverse effects. In particular, it can create conditions conducive to the development of disease. In cocoa-based SAFs (agroforestry systems), for example, while shading reduces the presence of pests such as mirids, it can accelerate pod rot. A study found a significant impact of shading on mirid bites and other types of damage [8]. This knowledge about the dual nature of shading effects in cocoa agroforestry systems is crucial. While the agroecological benefits of shading trees are well-documented [9], the specific effects of shading on the physicochemical and functional attributes of cocoa beans have not been studied in depth. This gap in knowledge is a key area for study, particularly in understanding how different degrees of shade influence the quality and characteristics of cocoa beans. Therefore, this study is aimed at investigating the nuances of how tree-induced shading in cocoa plantations affects the physicochemical and functional properties of cocoa beans. It focuses specifically on cocoa beans produced in Bonon, a region of significant importance in the national landscape of cocoa production in Côte d'Ivoire.

## 2. Materials and Methods

### 2.1. Study Site

**Figure 1:**
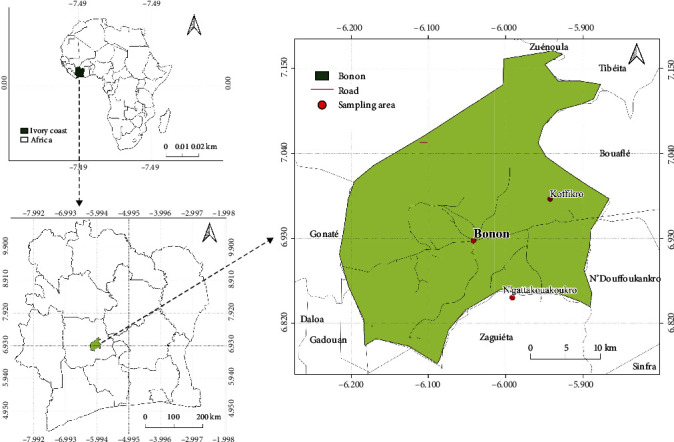
Study area and sampling sites. Top-left map: the continent of Africa with Côte d'Ivoire highlighted. Bottom-left map: a closer look at Côte d'Ivoire with a focus on Bonon locality. Right map: this is the most detailed map and zooms in on the subprefecture of Bonon within the Marahoué region. Bonon is indicated by a green square. Two villages, N'gattakouakoukro and Koffikro, where the cocoa plantations were sampled, are marked with red dots.

This research was conducted in the subprefecture of Bonon, nestled within the lush Marahoué region in the central-western part of Côte d'Ivoire. Bonon, a region rich in agricultural diversity and known for its vibrant cocoa production, is geographically positioned between 6°45′0^″^ and 7°10′0^″^ North latitude and 5°52′0^″^ and 6°14′0^″^ West longitude (Figure 1). This area spans approximately 520 square kilometers, presenting a diverse terrain that is quintessential for cocoa cultivation [10]. The climate of Bonon is predominantly of the Guinean type, characterized by its distinct wet and dry seasons. This climatic pattern plays a pivotal role in shaping the agricultural practices and crop yield in the region. The average temperature from January to June 2022 was 28.05°C. The lushness and fertility of the land, coupled with the favorable climate, make it an ideal locale for cocoa farming, a key economic activity in the area. For the purposes of this study, two villages within this region were selected: N'gattakouakoukro and Koffikro (Figure 1). These villages, emblematic of the region's agricultural prowess, are home to some of the most productive cocoa plantations in the subprefecture. Three such plantations, two located in Koffikro and one in N'gattakouakoukro, were chosen as the primary research sites for this study. These plantations were selected based on a set of criteria that included their size, yield, and the varying degrees of shade provided by the native tree cover. In each of the plots sampled, the various trees encountered were fruit trees such as *Mangifera indica* L, *Persea americana* Mill, and a forest type tree, notably *Tectona grandis*.

### 2.2. Vegetal Materials

*Theobroma cacao* L., the botanical denomination, hails from the Malvaceae family and is native to the deep tropical regions of Mesoamerica. It is a species rich in history and cultural significance, revered since ancient times for its unique flavor and purported medicinal properties. The cacao tree bears fruits, known as cacao pods, which are harvested for their seeds—cocoa beans. These beans undergo a series of postharvest processes that are pivotal in defining the flavor profile of chocolate [11]. The cocoa pods were harvested at the stage of physiological maturity, which can be appreciated by the size and the change in colour of the pods from green to yellow.

### 2.3. Sample Collections

The cocoa pods were harvested during the minor harvest season, specifically from April to June 2022. The sample collection method began by selecting three plantations based on the availability of ripe and mature cocoa pods. Details of the characteristics of the chosen plantations are recorded in Table 1. In each selected field, three subplots of 25 m × 25 m were established based on the number of trees present and the canopy density. Indeed, the different levels of shade were defined using a method slightly modified from Gala et al. [12]. A correlation was established between the number of trees present, canopy density, and the level of shade. Based on the density of tree shade, three distinct areas were identified within each subplot: shaded, moderately shaded, and sunny. A shaded area is defined as a zone with a dense tree (one or more) canopy that significantly restricts light penetration. In contrast, a semishaded area is characterized by a tree (one or more) with a smaller canopy, allowing a substantial amount of light to pass through. A sunny area, on the other hand, lacks tree cover and is thus fully exposed to sunlight.

### 2.4. Postharvest Treatment for Commercial Cocoa Production

The postharvest handling process leading to the production of commercial cocoa beans was carried out in three stages (pod breaking, fermentation, and drying). The pod breaking was done 3 days after harvest using a machete. Then, banana leaves were used as a base for fermenting the beans over a six-day period following the method recommended by Ban Koffi et al. [13]. This fermentation technique involves placing the beans on a slope on banana leaves. Finally, the fermented beans are dried for four days on a cement surface.

### 2.5. Determining the Commercial Quality of the Beans

#### 2.5.1. Graining

The quality of the fermented and dried beans was evaluated by graining. Graining is an important quality parameter [14]. To determine the graining, 100 grams of beans are weighed, and the beans are counted. With the average weight of a good bean being 1 gram, the quality of the graining will depend on the number of beans. Thus, the fewer the number of beans obtained for 100 g, the better the graining [15].

#### 2.5.2. Shell Percentage in Beans

In West Africa, the shell generally represents about 11 to 12% of the total weight of the bean, a ratio considered the norm for evaluating cocoa [15]. The proportion of shells in the cocoa beans is determined according to the CAOBISCO/ECA/FCC method [15]. This method involves weighing 100 beans followed by the removal of the shells. The percentage of shells in the beans is then calculated using the following formula: Percentage of shells = (mass of shells/mass of 100 beans) × 100.

### 2.6. Biochemical Characterization of Fermented Cocoa Beans

The pH and ash content were determined according to the method [16]. The water content was determined according to the AOAC method [16]. The titratable acidity was determined by titration with a sodium hydroxide solution (0.1 N) in the presence of phenolphthalein according to the method [16]. The lipid content was estimated by the AOAC method [17]. The BIPEA method [18] was used to determine the crude protein content through nitrogen content. The total carbohydrate rate was assessed according to the method described by FAO/WHO/UNU [19]. The energy value was calculated using the specific Atwater and Benedict coefficients [20], which take into account proteins, lipids, and carbohydrates. Finally, the vitamin C content of the beans was determined according to the iodine method described by Kolthoff and Sandell [21].

### 2.7. Functional Properties of Fermented Cocoa Beans

The water absorption capacity (WAC) of the cocoa beans was determined according to the Sosulski method [22]. The oil absorption capacity of the samples (OAC) was measured according to the method of Lin et al. [23]. Emulsifying activity (EA) and emulsion stability (ES) were determined by the method of Neto et al. [24]. Finally, the foaming capacity (FC) and stability (FS) were evaluated by the method described by Lin et al. [23].

### 2.8. Statistical Analysis

The data entry for the study was carried out using Microsoft Excel 2016. The R studio software was used to determine the means and standard deviations. Furthermore, a one-way analysis of variance was performed to compare the means and appreciate the existence of statistically significant differences between the samples.

## 3. Results and Discussion

### 3.1. Commercial Quality of Cocoa Beans from Shaded Area Plantations

The study's assessment of the commercial quality of cocoa beans from the Bonon region primarily focused on graining and shell percentage. The morphological characteristics examined showed variations depending on the level of shade (Table 2). Samples from shaded areas (FS1, FS2, and FS3) exhibited a graining quality, contrary to other samples whose rates exceeded the tolerable limit of 105. The shell percentages varied from 10.10 ± 0.49% to 24.01 ± 8.24%. Similar to graining, only samples from shaded areas (FS1, FS2, and FS3) presented shell percentages close to the norm (11-12%) required for exporting West African cocoa. This suggests that shade favors a good proportion of almonds, thus reducing the shell content. The superior bean quality observed in shaded areas can be attributed to shade-increasing bean weight, particularly the number of beans per 100 grams. This finding underscores the benefit of consistent shade in maintaining the quality and productivity stability of cocoa beans [25]. Additionally, Kouadio et al. [26] note that shade in agroforestry systems decreases and delays cocoa tree flowering. Shade alters the amount of light, temperature, and air movement in cocoa plantations, directly affecting photosynthesis, growth, and yield of cocoa trees. Moreover, excessive shade creates a more humid microclimate, promoting the proliferation of diseases such as brown rot, particularly affecting production [27]. In West African primary cocoa culture, the shell represents 11 to 12% of the total bean weight [14]. In this study, only shell percentages observed in samples from shaded areas (10.10 ± 0.49 for FS1, 10.43 ± 0.17 for FS2, and 10.00 ± 0.19 for FS3) conformed to the standards. This suggests that shade favors a good proportion of almonds, thereby reducing shell content. These samples could be readily used in the chocolate industry due to their low shell rates. However, the high shell percentages in other samples (semishaded and sunny areas) associated with poor graining indicate a lesser amount of almonds and poor commercial quality, even if this offers more protection to the nib [13, 15].

### 3.2. Biochemical Quality of the Beans Analysed

**Figure 2:**
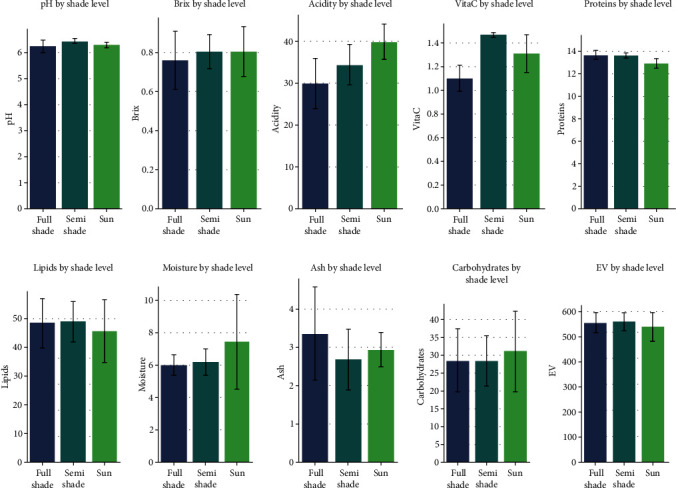
Physicochemical characterization of cocoa beans related to shade levels (full shade, semishade, and sun). pH: potential of hydrogen; Brix: Brix degree; acidity: total acidity; VitaC: vitamin C; EV: energy value.

The evaluation of the biochemical quality of the beans showed significant variation in biochemical composition among all samples based on their geographic origin and shade level (Table 3). To better appreciate the impact of the level of shade on the biochemical composition, a compilation of data by zone (shaded, semishaded, and sunny) was performed by calculating the averages of each parameter (Figure 2). The use of analysis of variance (ANOVA) revealed, overall, that the levels of pH, titratable acidity, proteins, and vitamin C are significantly affected by the level of shade, with respective *p* values of 0.048, 0.0028, 0.00053, and 4.10^−6^. Thus, nutritionally, we observed that the lowest protein contents were found in sunny areas (S1, 12.65%). Furthermore, our results suggest that beans from shaded areas tend to display lower rates of vitamin C. However, the levels of lipids and carbohydrates, although occasionally higher (respectively, in SS2 and S1), do not seem to depend on the variation in the shade level.

The biochemical analysis of cocoa samples from different zones (shaded, semishaded, and full sun) revealed significant variations in their composition, raising significant implications for the cocoa industry and its by-products.

The results highlight the impact of shade on the pH of the beans, a crucial variable in the quality of final products [28]. Moreover, shade can significantly influence product acidity, interacting with climatic conditions and altering crop sensitivity to soil acidity [29]. Only samples from the shaded area had a pH closer to the ISO standards [30], suggesting optimal potential for bean fermentation and drying. Shade tree species can significantly affect the soil biogeochemistry, which in turn influences the pH of the soil and potentially the beans grown in it [31]. This acidity could also be exploited in the cosmetic industry, given its compatibility with the skin's pH values (5.2 to 7) [32]. Regarding protein content, the lowest levels were observed in beans from sunny areas, suggesting a correlation between direct sun exposure and a decrease in protein content in cocoa beans. These observations align with other studies stating that total nitrogen content increases in plots as the percentage of shade increases [33]. This assertion further reinforces the importance of shade in developing the nutritional properties of beans. Furthermore, beans from shaded areas (rich in proteins) would be beneficial in formulating products such as protein shakes or protein bars for consumers seeking protein-rich foods [34].

Vitamin C, as an essential antioxidant, plays a crucial role in the nutritional quality of cocoa beans. The results obtained for vitamin C content are consistent with other research confirming a positive correlation between this vitamin and light intensity [35]. This underscores the importance of shade in regulating vitamin C levels, which could have direct implications on the quality of cocoa-based final products.

In this study, moisture content, lipids, carbohydrates, and energy value showed no significant variations related to shade. According to some authors, the fluctuation of these parameters is related to the variety of beans, the cultivated soil, and fermentation.

### 3.3. Functional Properties of Cocoa Beans

**Figure 3:**
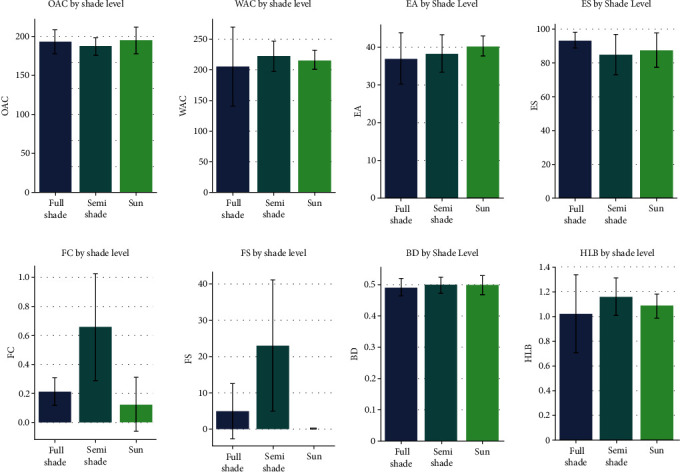
Functional characterization of cocoa beans related to shade levels (full shade, semishade, and sun). WAC: water absorption capacity; OAC: oil absorption capacity; EA: emulsifying activity; ES: emulsion stability; FC: foaming capacity; FS: foam stability; BD: block density; HLB: hydrophilic lipophilic balance.

The present study also examined the impact of shade density on the functional properties of cocoa beans, defining their capacities in industrial applications. The functional properties of cocoa beans were determined (Table 4). The statistical analysis of results reveals that only certain parameters such as bulk density, foam stability, foaming capacity, and emulsifying activity show significant differences between samples. The highest values for these parameters were observed in samples SS3, SS1, and FS2, respectively. The analysis of variance (ANOVA) based on the classification of samples into three groups (shaded, semishaded, and sunny) shows that only the foaming capacity (*p* value = 0.000043) and foam stability (*p* value = 0.000115) present *p* values below 0.05. These results indicate that shade levels significantly influence these parameters, with foaming capacity and stability being more pronounced in the semishaded zone (Figure 3). The high foaming capacity observed in semishaded areas suggests a possible involvement of globular protein rates [36]. These findings corroborate previous works by Suresh and Samsher [37], which emphasize that good foaming ability is related to flexible protein molecules, notably reducing surface tension. Concurrently, foam stability in these areas is attributed to an increase in proteins, enabling them to withstand gravitational and mechanical stresses [38]. However, it is essential to note that foam stability is also influenced by external factors such as pH and protein-polysaccharide complexes at the foam interfaces [39].

These results offer crucial perspectives for the food industry, indicating that beans from semishaded areas are ideal for formulating products requiring stable foam, such as chocolate mousse, whipped beverages, and certain desserts [38]. The capacity and stability of the foam of cocoa beans grown under moderate shade can thus play a key role in the perceived quality and application of these beans in the food industry.

On the other hand, parameters such as WAC (water absorption capacity), OAC (oil absorption capacity), EA (emulsifying activity), ES (emulsion stability), BD (bulk density), and HLB (hydration/lipid ratio) although important, seem to be relatively insensitive to shade. According to the writings of some authors (Suresh and Samsher [37]), these characteristics are influenced by factors such as protein conformation, amino acid composition, carbohydrate content, as well as surface polarity or hydrophobicity, highlighting the complexity of biochemical interactions within cocoa beans.

This study thus sheds light on the impact of shade on the functional properties of cocoa beans, enriching our understanding of the complex relationships between the growth environment and the biochemical characteristics. These nuances are essential for guiding the development of innovative and promising food products, taking into account the specificities of cocoa bean cultivation.

## 4. Conclusion

The findings of this research underscore the significant role that tree shade plays in cocoa plantations, particularly in terms of the commercial, nutritional, and functional attributes of cocoa beans. In areas with adequate shading, cocoa beans exhibited enhanced physical qualities, including better graining and shell composition. The study also revealed that shade conditions notably affect the pH, protein content, and levels of vitamin C in the beans. A notable observation was the increased protein concentration in beans from shaded areas, indicating a possible inverse relationship between direct sunlight exposure and protein levels in cocoa beans. Conversely, beans from less shaded, sunnier areas showed higher vitamin C content. Additionally, the investigation into functional properties revealed that beans from moderately shaded areas possessed superior foaming capacity and stability, an attribute particularly beneficial for specific culinary applications such as chocolate mousse preparation. These insights highlight the critical influence of shade management on the quality of cocoa beans and its subsequent impact on the processing industry. The results of this study not only contribute valuable knowledge to the field of food biochemistry but also pave the way for further research in sustainable agricultural practices.

## Figures and Tables

**Table 1:** Characteristics of selected fields.

Locality	Field	Village	Cocoa variety	Age group of field	Area
Bonon	1	Koffikro	Ghana/Cameroon	5-15 years	7.1 Ha
Bonon	2	Koffikro	All types	5-15 years	3.5 Ha
Bonon	3	N'gattakouakoukro	All types	5-15 years	2.02 Ha

**Table 2:** Average number of beans per 100 g and percentage in shells.

Samples	Average number of beans	Shell percentage
FS1	105 ± 2.64a	10.10 ± 0.49a
SS1	124 ± 3.21bc	15.68 ± 2.83ac
S1	135 ± 4.72c	19.77 ± 0.35bc
FS2	99 ± 0.57aa	10.43 ± 0.17a
SS2	121 ± 4.04b	12.45 ± 1.53ab
S2	123 ± 4b	18.07 ± 1.25ac
FS3	105 ± 1.52a	10.00 ± 0.19a
SS3	125 ± 6.02bc	24.01 ± 8.24c
S3	130 ± 4bc	18.24 ± 0.016ac

Values with different alphabetical letters on the same line are statistically different (*p* < 0.05). FS1: full shade field 1; SS1: semishade field 1; S1: sun field 1; FS2: full shade field 2; SS2: semishade field 2; S2: sun field 2; FS3: shade field 3; SS3: semishade field 3; S3: sun field 3.

**Table 3:** Physicochemical properties of cocoa beans from different fields.

Sample	pH	Total acidity (meq/100 g)	Ash (%)	Brix degree	Vitamin C **(**mg/100 g)	Moisture (%)	Lipids (%)	Carbohydrates (%)	Proteins (%)	Energy value (kcal)
FS1	6.29 ± 0.01c	32.66 ± 7.2ab	2.56 ± 0.51a	0.83 ± 0.05ab	1.08 ± 0.03ab	6.65 ± 0.54a	42.08 ± 0.18ab	35.93 ± 0.16ab	13.30 ± 0.30ac	573.53 ± 1.02ab
SS1	6.49 ± 0.01e	32.21 ± 6.2ab	2.24 ± 0.57a	0.83 ± 0.05ab	1.45 ± 0.01d	6.73 ± 1.45a	44.78 ± 0.24ac	32.34 ± 2.27ab	13.88 ± 0.2c	587.98 ± 7.57d
S1	6.42 ± 0.02de	38.00 ± 6ab	3.25 ± 0.54ab	0.90 ± .0.0b	1.52 ± 0.02d	6.84 ± 0.92a	31.27 ± 3.44a	45.96 ± 3.81b	12.65 ± 0.2a	515.99 ± 17.06d
FS2	5.95 ± 0.04a	25.28 ± 8.6a	2.64 ± 0.57a	0.86 ± 0.05ab	1.23 ± 0.05c	5.45 ± 0.37a	46.58 ± 10.55bc	31.49 ± 10.26ab	13.82 ± 0.6c	600.56 ± 53.20b
SS2	6.52 ± 0.01e	31.11 ± 6.7ab	2.94 ± 1.00ab	0.73 ± 0.11ab	1.47 ± 0.02d	5.85 ± 0.32a	58.04 ± 4.49c	19.62 ± 4.79a	13.53 ± 0.2bc	655.01 ± 21.82d
S2	6.17 ± 0.05b	42.49 ± 4.4b	2.96 ± 0.01ab	0.66 ± 0.15ab	1.18 ± 0.04bc	6.91 ± 2.57a	56.63 ± 2.85bc	23.63 ± 10.24a	12.71 ± 0.1ab	655.15 ± 15.42bc
FS3	6.51 ± 0.02e	31.68 ± 1.8ab	4.87 ± 0.99b	0.60 ± 0.17a	0.98 ± 0.02a	5.89 ± 0.51a	56.73 ± 5.48bc	18.61 ± 4.22a	13.88 ± 0.2c	640.60 ± 32.09b
SS3	6.32 ± 0.05cd	33.93 ± 1.9ab	2.86 ± 0.93ab	0.86 ± 0.05ab	1.46 ± 0.02d	5.94 ± 0.05a	44.78 ± 0.28ac	33.36 ± 3.8ab	13.59 ± 0.1c	585.90 ± 12.08d
S3	6.29 ± 0.03c	38.94 ± 3.2ab	2.62 ± 0.54a	0.86 ± 0.05ab	1.22 ± 0.05c	8.56 ± 5.26a	48.67 ± 3.67bc	26.64 ± 1.43a	13.47 ± 0.36ac	598.59 ± 37.19c

Values with different alphabetical letters on the same line are statistically different (*p* < 0.05). FS1: full shade field 1; SS1: semishade field 1; S1: sun field 1; FS2: full shade field 2; SS2: semishade field 2; S2: sun field 2; FS3: shade field 3; SS3: semishade field 3; S3: sun field 3.

**Table 4:** Functional properties of cocoa beans from different fields.

Sample	OAC (%)	WAC (%)	EA (%)	ES (%)	FC (%)	FS (%)	BD (g/cm^3^)	HLB
FS1	210.80 ± 5.30a	237.44 ± 7.59a	36.87 ± 0.89ac	95.31 ± 4.76a	0.11 ± 0.00a	15.00 ± 5a	0.45 ± 0.0a	1.12 ± 0.03a
SS1	197.56 ± 10.74a	199.35 ± 36.91a	40.02 ± 4.78ac	89.98 ± 4.37a	0.82 ± 0.27b	35.55 ± 3.84b	0.49 ± 0.00ab	1.00 ± 0.16a
S1	193.83 ± 3.94a	200.14 ± 4.36a	38.79 ± 1.25ac	93.65 ± 10.99a	0.37 ± 0.13ab	0.00a	0.51 ± 0.05b	1.03 ± 0.005a
FS2	179.68 ± 15.24a	223.95 ± 5.92a	44.47 ± 5.7c	92.35 ± 3.32a	0.22 ± 0.00a	0.00a	0.50 ± 0.01ab	0.83 ± 0.6a
SS2	188.23 ± 16.02a	228.26 ± 4.94a	33.4 ± 5.34ab	91.08 ± 8.82a	0.29 ± 0.12ab	0.00a	0.47 ± 0.00ab	1.21 ± 0.11a
S2	206.91 ± 33.89a	231.50 ± 16.37a	41.64 ± 2.29bc	88.72 ± 11.36a	0.00a	0.00a	0.48 ± 0.05ab	1.13 ± 0.16a
FS3	201.29 ± 7.20a	222.16 ± 5.4a	29.86 ± 3.11a	92.67 ± 7.14a	0.29 ± 0.11ab	0.00a	0.51 ± 0.02ab	1.10 ± 0.06a
SS3	187.75 ± 7.79a	238.13 ± 10.97a	41.50 ± 1.64ac	73.94 ± 16.25a	0.84 ± 0.48b	33.33 ± 16.66b	0.52 ± 0.00b	1.26 ± 0.06a
S3	196.43 ± 7.35a	216.20 ± 6.58a	40.74 ± 3.96bc	81.00 ± 10.09a	0.00a	0.00a	0.49 ± 0ab	1.10 ± 0.06a

Values with different alphabetical letters on the same line are statistically different (*p* < 0.05). FS1: full shade field 1; SS1: semishade field 1; S1: sun field 1; FS2: full shade field 2; SS2: semishade field 2; S2: sun field 2; FS3: shade field 3; SS3: semishade field 3; S3: sun field 3; WAC: water absorption capacity; OAC: oil absorption capacity; EA: emulsifying activity; ES: emulsion stability; FC: foaming capacity; FS: foam stability; BD: block density; HLB: hydrophilic lipophilic balance.

## Data Availability

The data supporting the conclusions of this study are included within the article.
